# Health-Related Quality of Life Outcomes in Patients with Myelodysplastic Syndromes with Ring Sideroblasts Treated with Luspatercept in the MEDALIST Phase 3 Trial

**DOI:** 10.3390/jcm11010027

**Published:** 2021-12-22

**Authors:** Esther Natalie Oliva, Uwe Platzbecker, Guillermo Garcia-Manero, Ghulam J. Mufti, Valeria Santini, Mikkael A. Sekeres, Rami S. Komrokji, Jeevan K. Shetty, Derek Tang, Shien Guo, Weiqin Liao, George Zhang, Xianwei Ha, Rodrigo Ito, Jennifer Lord-Bessen, Jay T. Backstrom, Pierre Fenaux

**Affiliations:** 1 Grande Ospedale Metropolitano Bianchi Melacrino Morelli, 89124 Reggio Calabria, Italy; 2 Medical Clinic and Policlinic 1, Hematology and Cellular Therapy, Leipzig University Hospital, 04103 Leipzig, Germany; uwe.platzbecker@medizin.uni-leipzig.de; 3 The University of Texas MD Anderson Cancer Center, Houston, TX 77030, USA; ggarciam@mdanderson.org; 4 Department of Haemato-Oncology, King’s College London, London SE5 9RS, UK; ghulam.mufti@kcl.ac.uk; 5 MDS Unit, Azienda Ospedaliero Universitaria Careggi, University of Florence, 50134 Florence, Italy; valeria.santini@unifi.it; 6 Sylvester Comprehensive Cancer Center, University of Miami, Miami, FL 33136, USA; msekeres@med.miami.edu; 7 Moffitt Cancer Center, Tampa, FL 33612, USA; Rami.Komrokji@moffitt.org; 8 Bristol Myers Squibb, Princeton, NJ 08540, USA; jeevan.shetty@bms.com (J.K.S.); derek.tang@bms.com (D.T.); george.zhang@bms.com (G.Z.); xianwei.ha@bms.com (X.H.); rodrigo.ito@gmail.com (R.I.); Jennifer.Lord-Bessen@bms.com (J.L.-B.); 9 Evidera, Waltham, MA 02451, USA; shien.guo@evidera.com (S.G.); weiqin.liao@evidera.com (W.L.); 10 Acceleron Pharma, Cambridge, MA 02139, USA; jbackstrom@acceleronpharma.com; 11 Service d’Hématologie Séniors, Hôpital Saint-Louis, Assistance Publique des Hôpitaux de Paris (AP-HP), 75010 Paris, France; pierre.fenaux@aphp.fr; 12 Senior Hematology Department, Saint Louis Hospital, Paris 7 University, 75013 Paris, France

**Keywords:** transfusion dependence, quality of life, myelodysplastic syndromes, luspatercept

## Abstract

Patients with myelodysplastic syndromes (MDS) often experience chronic anemia and long-term red blood cell transfusion dependence associated with significant burden on clinical and health-related quality of life (HRQoL) outcomes. In the MEDALIST trial (NCT02631070), luspatercept significantly reduced transfusion burden in patients with lower-risk MDS who had ring sideroblasts and were refractory to, intolerant to, or ineligible for prior treatment with erythropoiesis-stimulating agents. We evaluated the effect of luspatercept on HRQoL in patients enrolled in MEDALIST using the EORTC QLQ-C30 and the QOL-E questionnaire. Change in HRQoL was assessed every 6 weeks in patients receiving luspatercept with best supportive care (+ BSC) and placebo + BSC from baseline through week 25. No clinically meaningful within-group changes and between-group differences across all domains of the EORTC QLQ-C30 and QOL-E were observed. On one item of the QOL-E MDS-specific disturbances domain, patients treated with luspatercept reported marked improvements in their daily life owing to the reduced transfusion burden, relative to placebo. Taken together with previous reports of luspatercept + BSC reducing transfusion burden in patients from baseline through week 25 in MEDALIST, these results suggest luspatercept may offer a treatment option for patients that reduces transfusion burden while providing stability in HRQoL.

## 1. Introduction

Myelodysplastic syndromes (MDS) are a heterogeneous group of clonal hematopoietic neoplasms characterized by ineffective hematopoiesis, progressive cytopenias, and risk of progression to acute myeloid leukemia [1,2]. At diagnosis, about 90% of patients with MDS experience anemia [3] which can lead to symptoms of fatigue, cardiac morbidity, and negative impacts on health-related quality of life (HRQoL) [4,5,6,7,8,9].

Treatments for patients with lower-risk MDS are largely aimed at mitigating anemia and thereby improving their HRQoL [2,10]. For patients who are refractory to erythropoiesis-stimulating agents (ESAs), anemia is commonly managed with frequent red blood cell (RBC) transfusions [2]. Specifically, about 40% of patients with MDS are dependent on treatment with regular RBC transfusions as part of their supportive care regimen [1,3,10]. Of note, treatment with RBC transfusions can provide transient relief in anemia-related symptoms, particularly fatigue and dyspnea, which can be associated with short-term improvements in HRQoL measures such as physical, role, and social functioning [11,12]. Long-term dependence on RBC transfusions, however, is associated with poor prognosis and can cause complications due to iron overload including hepatic and cardiac organ failure; these conditions can further exacerbate negative impacts on patients’ HRQoL [13,14,15,16,17].

For patients with transfusion-dependent lower-risk MDS, a therapeutic option that can address the underlying causes of chronic anemia and reduce transfusion burden is imperative, particularly for patients who are refractory to, intolerant to, or ineligible for treatment with ESAs. Luspatercept is a first-in-class erythroid maturation agent providing clinically meaningful reduction in transfusion burden in patients with transfusion-dependent anemia due to lower-risk MDS [18,19].

The phase 3 MEDALIST trial (NCT02631070) compared treatment with luspatercept and best supportive care (+ BSC) to placebo + BSC in patients with transfusion-dependent anemia due to lower-risk MDS. In the first 24 weeks of the trial, transfusion independence for ≥8 weeks was observed in 38% of patients in the luspatercept + BSC arm and only 13% of patients in the placebo + BSC arm. Furthermore, a greater proportion of patients (28%) in the luspatercept + BSC arm achieved transfusion independence for ≥12 weeks compared with 8% of patients in the placebo + BSC arm [18]. With this reduction in transfusion burden, however, the impact of luspatercept + BSC on patients’ HRQoL has not yet been reported. In the present analysis, we aimed to evaluate the effect of luspatercept + BSC, relative to placebo + BSC, on HRQoL in patients treated for lower-risk MDS from baseline through week 25 in the MEDALIST trial.

## 2. Methods

### 2.1. Study Design

**Figure 1:**
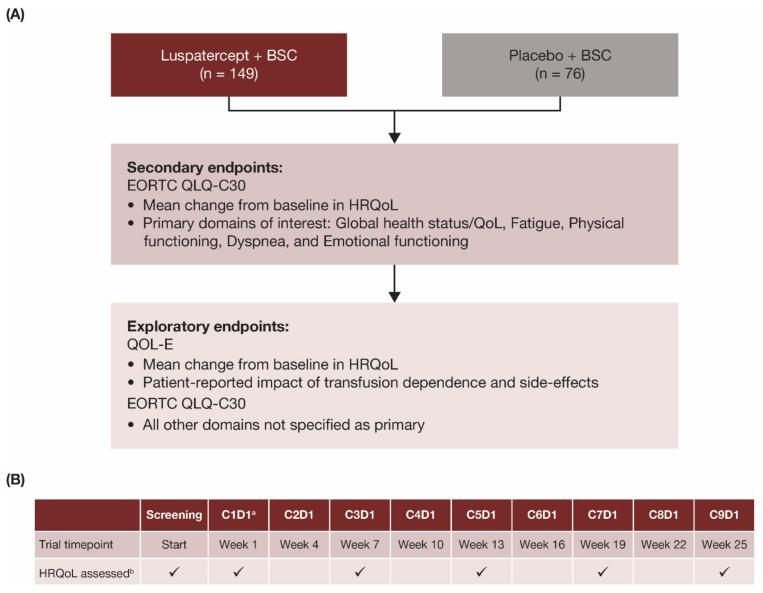
(**A**) Endpoints and (**B**) assessment schedule for patient-reported HRQoL outcomes in the MEDALIST trial. ^a^ Baseline visit. ^b^ HRQoL assessed with EORTC QLQ-C30 and QOL-E instruments on days indicated with checkmark (✓). BSC, best supportive care; C, cycle; D, day; EORTC QLQ-C30, European Organisation for Research and Treatment of Cancer’s Core Quality of Life Questionnaire; HRQoL, health-related quality of life; MDS, myelodysplastic syndromes; QoL, quality of life; QOL-E, Quality of Life Assessment in MDS questionnaire.

In the double-blind, placebo-controlled, randomized, phase 3 MEDALIST trial (NCT02631070), patients were randomized in a 2:1 ratio to receive luspatercept (1.0–1.75 mg/kg) or placebo subcutaneously every 3 weeks for 24 weeks, plus BSC including RBC transfusions given at the investigator’s discretion [18]. The primary endpoint of the MEDALIST trial was transfusion independence for ≥8 weeks during weeks 1–24 and the key secondary endpoint was transfusion independence for ≥12 weeks assessed during weeks 1–24 and weeks 1–48, as reported previously [18]. Effects of luspatercept versus placebo on patient-reported outcomes (PRO) in HRQoL were evaluated as secondary and exploratory endpoints in the MEDALIST trial (Figure 1A).

HRQoL data were collected prior to the administration of study drugs at each scheduled visit, independent of RBC transfusion events, using electronic tablets as the primary method and paper-and-pencil as a supplemental method. Specifically, HRQoL was assessed at screening, cycle 1 day 1 (C1D1, baseline), and every other 3-week cycle (Figure 1B) during the primary treatment phase to week 25 (which marked the completion of 24 calendar weeks after the date of the first dose, regardless of dose delay).

### 2.2. Patient Selection

Patients with a Revised International Prognostic Scoring System (IPSS-R) score of very low-risk, low-risk, or intermediate-risk MDS with ring sideroblasts who had been receiving regular RBC transfusions were included, as described previously [18]. Eligible patients were ≥18 years of age; had a documented diagnosis of MDS; and were refractory to, intolerant to, or ineligible (serum erythropoietin > 200 U/L) for ESA treatment.

Patients were included if they received an average of ≥2 units/8 weeks of packed RBC transfusions during the 16 weeks before randomization. Patients with hemoglobin (Hb) levels ≤ 10 g/dL at the time of or within 7 days prior to administration of an RBC transfusion were included; RBC transfusions administered when Hb levels were >10 g/dL and/or RBC transfusions administered for elective surgery did not qualify as a required transfusion to meet eligibility criteria. Patients were excluded if they had a consecutive 56-day period (≥8 weeks) that was RBC transfusion-free during the 16 weeks before randomization.

The intent-to-treat (ITT) population included all subjects who were randomized in the study, whereas the HRQoL-evaluable population included all subjects in the ITT population who completed the HRQoL assessments at the baseline visit (or at the screening visit if assessment at the C1D1 visit was not completed, captured, or available) and had at least one post-baseline assessment visit.

### 2.3. HRQoL Assessments

HRQoL was assessed using the European Organisation for Research and Treatment of Cancer’s Core Quality of Life Questionnaire (EORTC QLQ-C30) version 3.0 [20] and the Quality of Life assessment in MDS questionnaire (QOL-E) version 3.0 [21]. Primary domains of interest on the EORTC QLQ-C30 were global health status/QoL, physical functioning, emotional functioning, fatigue, and dyspnea, as these were considered the most clinically relevant to patients with MDS (Figure 1A). All other domains on the EORTC QLQ-C30 were assessed as exploratory domains of interest: role functioning, cognitive functioning, social functioning, nausea/vomiting, pain, insomnia, appetite loss, constipation, diarrhea, and financial difficulties. Scores ranged from 0 to 100. In the global health status/QoL and functioning domains, higher scores represent better QoL, whereas in all other domains, higher scores represent worse QoL.

The QOL-E questionnaire, an MDS-specific assessment, was of exploratory interest. QOL-E domains of physical well-being, functional well-being, social and family life, sexual well-being, fatigue, and MDS-specific disturbances were included in the exploratory analyses; specific patient-reported impact of transfusion dependence and treatment side effects were reported using the MDS-specific disturbances domain. QOL-E summary scales included the treatment outcome index (TOI), which was the summary of physical well-being, functional well-being, and MDS-specific disturbances domain scores; “General”, which was the summary of all domain scores except for MDS-specific disturbances; and “All”, which was the summary of all domain scores. Scores ranged from 0 to 100. Higher scores represent better QoL across all domains and summary scales.

### 2.4. Statistical Analyses

The data-cutoff date was 1 July 2019. Descriptive statistics of the baseline HRQoL domain scores of each PRO measure and key demographic and clinical characteristics were summarized by treatment group and overall for the HRQoL-evaluable populations. Continuous variables were summarized using means and standard deviations, while categorical variables were summarized using percentages.

Least-squares (LS) mean difference in change in domain scores from baseline to week 25 (clinical assessment visit) between luspatercept and placebo was determined using mixed-effects repeated-measures analysis. A minimal clinically important difference (MCID) within each treatment arm was defined as a ≥10-point change in score from baseline for all EORTC QLQ-C30 domains [22] and ≥0.5 standard deviations of the baseline domain score for all QOL-E domains and summary scales [23,24]. Differences in scores between luspatercept and placebo arms were considered clinically meaningful if the difference in the change from baseline between treatment arms exceeded the MCID threshold. All analyses were performed using SAS software (SAS Institute Inc., Cary, NC, USA), version 9.4 or above. The study was not powered to detect treatment differences in HRQoL endpoints.

## 3. Results

### 3.1. Patients

The ITT population consisted of a total of 229 patients who were randomized: 153 patients to luspatercept + BSC and 76 to placebo + BSC. The HRQoL-evaluable population, consisting of patients with baseline and at least one post-baseline EORTC QLQ-C30 assessment, was 149 patients in the luspatercept + BSC arm and 76 patients in the placebo + BSC arm. Baseline demographics and clinical characteristics of the HRQoL-evaluable population are shown in Table 1. For these 225 patients, the mean age was 70.6 years, 63.6% of patients were male, and 69.3% were white. Most patients (82.7%) had IPSS-R very low-risk or low-risk MDS and 16.9% had intermediate-risk MDS.

### 3.2. EORTC QLQ-C30 Assessment

EORTC QLQ-C30 questionnaire compliance rates among ITT patients remaining on treatment were similar between luspatercept + BSC (83.6–98.0%) and placebo + BSC (79.4–100.0%) treatment groups from baseline through week 25. Baseline scores were similar between luspatercept + BSC and placebo + BSC treatment groups; the overall baseline scores for each domain are shown in Table 2. At baseline, patients in the MEDALIST trial had a clinically meaningful worse HRQoL compared with the general population [25] in 5 of 15 EORTC QLQ-C30 domains: physical functioning, role functioning, social functioning, fatigue, and dyspnea (Table 2). MEDALIST patients were similar at baseline in HRQoL to patients with recurrent or metastatic cancer [26]; most EORTC QLQ-C30 domain scores for MEDALIST patients were within 10 points of the corresponding domain scores for patients with recurrent or metastatic cancer.

**Figure 2:**
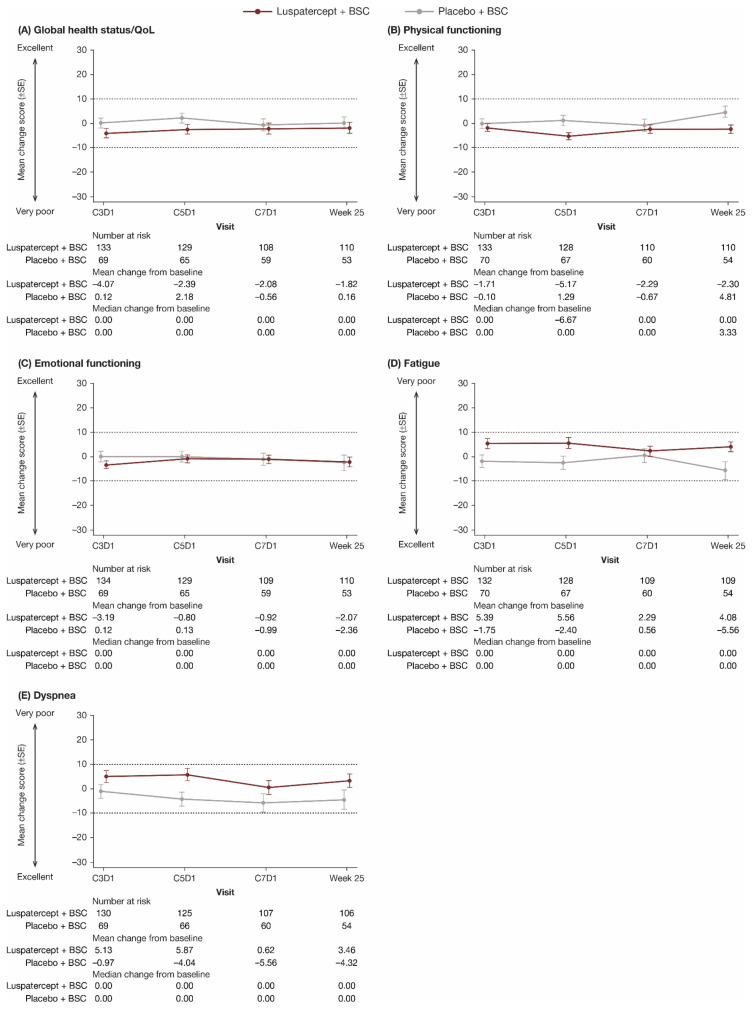
Observed mean change from baseline in patient-reported (**A**) global health status/QoL, (**B**) physical functioning, (**C**) emotional functioning, (**D**) fatigue, and (**E**) dyspnea scores through week 25 on the EORTC QLQ-C30. Dashed lines indicate threshold for a clinically meaningful difference. In (**A**–**C**), higher scores represent better QoL; in (**D**,**E**), higher scores represent worse QoL. BSC, best supportive care; C, cycle; D, day; EORTC QLQ-C30, European Organisation for Research and Treatment of Cancer’s Core Quality of Life Questionnaire; QoL, quality of life; SE, standard error.

The primary EORTC QLQ-C30 domains of interest of global health status/QoL, physical functioning, emotional functioning, fatigue, and dyspnea are shown in Figure 2. All other domains are shown in Supplementary Figure S1. For each domain, through week 25, there was no clinically meaningful difference in mean change from baseline between and within the luspatercept + BSC and placebo + BSC groups in all EORTC QLQ-C30 domains. Longitudinal mixed-model analyses showed that the LS mean difference in change in all EORTC QLQ-C30 domain scores from baseline to week 25 between luspatercept + BSC and placebo + BSC was within the MCID (Table 3).

### 3.3. QOL-E Assessment

**Figure 3:**
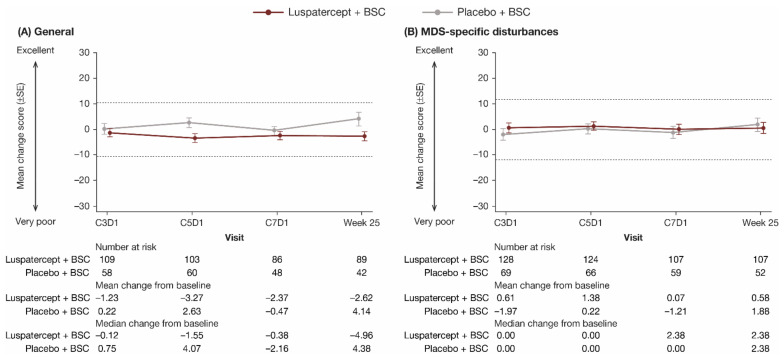
Observed mean change from baseline in patient-reported (**A**) General (summary of all domain scores except for MDS-specific disturbances) and (**B**) MDS-specific disturbances scores through week 25 on the QOL-E. Dashed lines indicate threshold for a clinically meaningful difference. BSC, best supportive care; C, cycle; D, day; MDS, myelodysplastic syndromes; QOL-E, Quality of Life Assessment in MDS questionnaire; SE, standard error.

QOL-E questionnaire compliance rates among ITT patients remaining on treatment were similar between luspatercept + BSC (82.8–98.7%) and placebo + BSC (77.9–100.0%) treatment groups from baseline through week 25. Baseline scores were similar between luspatercept + BSC and placebo + BSC treatment groups; the overall baseline scores for each domain and summary scales are shown in Table 4. Through week 25, there was no clinically meaningful difference in mean change from baseline between and within the luspatercept + BSC and placebo + BSC groups in all QOL-E domains. The General summary scale and MDS-specific disturbances domain are shown in Figure 3; all other domains and summary scales are shown in Supplementary Figure S2. Longitudinal mixed-model analyses showed that the LS mean difference in change in all QOL-E domain scores from baseline to week 25 between luspatercept + BSC and placebo + BSC was within the MCID (Table 5).

**Figure 4:**
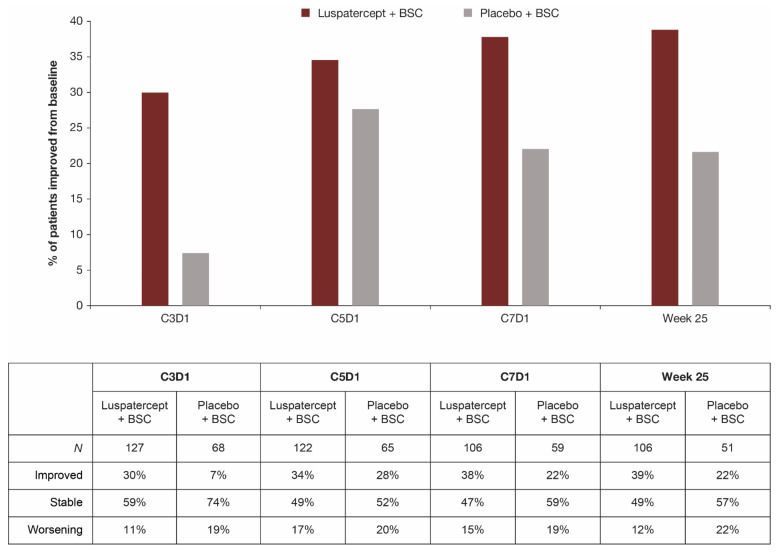
Patient-reported impact of transfusion burden by QOL-E from treatment initiation through week 25. Question from the QOL-E instrument: What effect of the disease disturbs your daily life? Being dependent on transfusions; response options: “No, not at all”, “A little bit”, or “Yes, extremely”. Responses at each timepoint were compared with responses at baseline to assess if patients had improved, remained stable, or experienced worsening. BSC, best supportive care; C3D1, cycle 3 day 1 (and similarly for C5D1 and C7D1); MDS, myelodysplastic syndromes; QOL-E, Quality of Life Assessment in MDS questionnaire.

The impact of treatment-related side effects on patients was comparable between luspatercept + BSC and placebo + BSC at week 25; 53.8% and 60.4% of patients receiving luspatercept and placebo, respectively, reported that side effects of the treatment did not disturb their daily life at all (Table 6). At week 25, a similar proportion of patients in the luspatercept + BSC (17.8%) and placebo + BSC (20.8%) group reported not being impacted by shortness of breath in the last week. The single item on the QOL-E that showed substantial difference between treatment groups was that regarding transfusion dependence; a greater proportion of patients in the luspatercept + BSC group relative to placebo + BSC consistently reported improvements in daily life from the impact of transfusion burden (Figure 4). Relative to baseline, the proportion of patients reporting a lower impact of transfusion dependence (improvement) on their daily life was 39% versus 22% in the luspatercept + BSC group versus the placebo + BSC group, respectively, at week 25. In contrast, the proportion of patients reporting a higher impact of transfusion dependence (worsening) on their daily life was 12% versus 22% in the luspatercept + BSC versus placebo + BSC, respectively.

## 4. Discussion

Luspatercept is a novel treatment option for patients with lower-risk MDS who have ring sideroblasts and require regular RBC transfusions [27]. It was previously reported that luspatercept + BSC significantly reduced RBC transfusion burden through week 25 in the MEDALIST trial [18]. In the present analysis, we found that this observed reduction in RBC transfusion burden with luspatercept occurred while maintaining HRQoL within a threshold that did not reflect a clinically meaningful change in patients through week 25 in the MEDALIST trial, based on EORTC QLQ-C30 and QOL-E assessments. Since baseline HRQoL of patients in the MEDALIST trial was similar to that observed in patients with recurrent or metastatic cancer (Table 2), it could be inferred that patients’ HRQoL remained comparable to that of other cancer patients through week 25. In other words, HRQoL did not worsen as luspatercept reduced RBC transfusions in patients, which has positive implications for patients’ HRQoL in the short and long term.

These findings have implications for patients with MDS who are transfusion-dependent and refractory to, intolerant to, or ineligible for treatment with ESAs. Long-term dependence on RBC transfusions may have detrimental clinical consequences, including iron overload and its associated complications of cardiac and hepatic organ failure [14,15,16], whereas cessation or reduction of RBC transfusions, when clinically indicated, may increase anemia-related symptoms and negatively impact HRQoL. Common anemia-related symptoms include fatigue, headache, chest pain, dizziness, and shortness of breath, which may lead to impaired mental alertness, physical weakness, loss of energy, and poor concentration [4,5,28,29,30]. These symptoms can have a profoundly negative impact on patients’ overall functioning and well-being [4,7,17].

In the present analysis, comparing luspatercept + BSC and placebo + BSC arms, no clinically meaningful differences in HRQoL through week 25 were observed within groups and between groups across all primary and exploratory domains of the EORTC QLQ-C30 and QOL-E assessments. This shows that in reducing RBC transfusions while on luspatercept, patients did not experience negative impacts on their HRQoL, contrary to what might be presumed, since transfusions provide transient relief from anemia-related symptoms. Furthermore, luspatercept significantly reduced patient-reported disturbances from RBC transfusions on daily life relative to placebo, likely owing to the reduced number of transfusions required in luspatercept-treated patients.

Although the analysis was not powered to detect statistically significant differences, the maintenance of HRQoL observed in the MEDALIST trial from the present analysis is consistent with historical clinical trials which evaluated changes in HRQoL in patients with lower-risk MDS [27,31,32,33,34,35,36,37,38,39,40,41]. Similar to these previous studies, our analysis showed that treatment with luspatercept did not worsen patients’ HRQoL.

There are some limitations in this analysis that should be noted. First, the majority of concepts covered by the EORTC QLQ-C30 (cancer-specific) and QOL-E (MDS-specific) instruments were not specific to the luspatercept treatment effect, particularly concepts directly capturing benefits from RBC transfusion reduction. Future research is needed in the development of specific HRQoL instruments in order to thoroughly understand the benefits of new treatments on transfusion burden in patients with MDS. Second, HRQoL endpoint data collection was set on a fixed schedule, independent of RBC transfusion events. RBC transfusions provide temporary relief and improvement in anemia-related symptoms that could positively impact HRQoL in the days following transfusion. However, these beneficial effects of RBC transfusions on HRQoL would likely have impacted the results of the placebo group, as these patients received more RBC transfusions, and the time between PRO administration and the preceding RBC transfusion was shorter. This could have further limited detection of HRQoL improvements when comparing the luspatercept and placebo arms. Third, patient Hb levels in the MEDALIST trial were maintained at a range where HRQoL changes may be insensitive to detection. It has been shown [42] that the incremental gain in HRQoL is largest when Hb levels are 10–12 g/dL, while HRQoL improvements appear minimal when Hb is below 10 g/dL. The average Hb level at baseline in the MEDALIST trial was 7.6 g/dL and the study design required a dose delay for patients whose Hb concentrations were ≥11.5 g/dL and who had a change in Hb level of ≥2 g/dL from the previous treatment cycle. However, despite these limitations, the results from our analysis suggest that, for patients with lower-risk MDS who have ring sideroblasts and are refractory to, intolerant to, or ineligible for ESAs, luspatercept may offer a treatment option that reduces transfusion burden while providing stability in HRQoL.

## 5. Conclusions

Luspatercept + BSC reduced RBC transfusion burden [18] and transfusion impact on the daily life of patients with MDS, while maintaining other aspects of HRQoL within a threshold that did not reflect a clinically meaningful change from baseline through week 25 in the MEDALIST trial.

## Figures and Tables

**Table 1:** Patient demographics and clinical characteristics at baseline of HRQoL-evaluable population.

Characteristic	Luspatercept + BSC (*n* = 149)	Placebo + BSC (*n* = 76)	Total (N = 225)
Age, years, mean (SD)	70.5 (8.7)	70.7 (10.9)	70.6 (9.4)
Age group, years, *n* (%)			
≤64	28 (18.8)	16 (21.1)	44 (19.6)
65–74	70 (47.0)	29 (38.2)	99 (44.0)
≥75	51 (34.2)	31 (40.8)	82 (36.4)
Sex, *n* (%)			
Male	93 (62.4)	50 (65.8)	143 (63.6)
Race, *n* (%)			
White	105 (70.5)	51 (67.1)	156 (69.3)
Black	1 (0.7)	0 (0.0)	1 (0.4)
Not collected	42 (28.2)	24 (31.6)	66 (29.3)
Other	1 (0.7)	1 (1.3)	2 (0.9)
IPSS-R risk, *n* (%)			
Very low or low	123 (82.6)	63 (82.9)	186 (82.7)
Intermediate	25 (16.8)	13 (17.1)	38 (16.9)
Missing	1 (0.7)	0 (0.0)	1 (0.4)
Prior ESA use, *n* (%)			
Yes	144 (96.6)	70 (92.1)	214 (95.1)
Transfusion burden, *n* (%)			
<4 RBCT units/8 weeks	44 (29.5)	20 (26.3)	64 (28.4)
4–5 RBCT units/8 weeks	40 (26.8)	23 (30.3)	63 (28.0)
≥6 RBCT units/8 weeks	65 (43.6)	33 (43.4)	98 (43.6)

ESA, erythropoiesis-stimulating agent; IPSS-R, Revised International Prognostic Scoring System; RBCT, red blood cell transfusion; SD, standard deviation.

**Table 2:** EORTC QLQ-C30 scores in the MEDALIST trial at baseline, in the general population, and in patients with recurrent or metastatic cancer.

EORTC QLQ-C30 Domain ^a^	Baseline Score in MEDALIST ^b^ Mean (SD) (N = 225)	Mean Score in General Population ^c^ (N = 11,343)	Mean Score in Patients with Recurrent/Metastatic Cancer ^d^ (N = 4812)
Global health status/QoL	58.3 (20.1)	67.1	56.3
Physical functioning	**66.3 (21.1)**	82.5	75.8
Role functioning	**65.1 (29.5)**	83.8	60.7
Cognitive functioning	82.1 (20.3)	87.2	80.5
Emotional functioning	76.9 (19.9)	81.6	68.7
Social functioning	**74.3 (27.8)**	89.1	70.5
Fatigue	**42.9 (24.6)**	24.9	41.8
Nausea/vomiting	5.0 (12.2)	2.5	13.1
Pain	18.9 (24.6)	23.2	33.7
Dyspnea	**35.7 (29.5)**	17.0	23.4
Insomnia	27.5 (30.8)	24.0	33.6
Appetite loss	14.4 (23.9)	6.8	28.2
Constipation	17.6 (27.0)	10.7	23.2
Diarrhea	8.9 (18.4)	6.2	10.7
Financial difficulties	11.0 (22.9)	7.6	16.2

^a^ Higher scores represent better QoL in global health status/QoL and functioning domains; higher scores in all other domains represent worse QoL. ^b^ For domain scores in bold, QoL was worse in MEDALIST patients compared with the general population; a difference of ≥10 points was considered a clinically meaningful difference. ^c^ From Nolte et al. [25]. The mean was re-weighted based on the age and gender distributions of the MEDALIST patients. ^d^ From Scott et al. [26]. EORTC QLQ-C30, European Organisation for Research and Treatment of Cancer’s Core Quality of Life Questionnaire; QoL, quality of life; SD, standard deviation.

**Table 3:** LS mean difference in change in EORTC QLQ-C30 domain scores from baseline to week 25 between luspatercept + BSC and placebo + BSC ^a^.

EORTC QLQ-C30 Domain	LS Mean (SE) Difference ^b^ at Week 25
Global health status/QoL	−3.76 (2.88)
Physical functioning	−7.13 (2.50)
Role functioning	−5.12 (4.15)
Cognitive functioning	1.62 (2.78)
Emotional functioning	−0.51 (2.89)
Social functioning	−3.12 (3.89)
Fatigue	6.76 (3.24)
Nausea/vomiting	−0.67 (1.96)
Pain	−1.07 (3.42)
Dyspnea	5.55 (3.87)
Insomnia	−1.04 (3.78)
Appetite loss	0.32 (3.73)
Constipation	3.80 (3.22)
Diarrhea	−0.86 (2.62)
Financial difficulties	0.58 (2.48)

^a^ Data from longitudinal mixed model analyses. ^b^ Differences in scores between luspatercept and placebo arms were considered clinically meaningful if the difference in the change from baseline between treatment arms exceeded the MCID threshold. MCID was defined as a ≥10-point difference. For global health status/QoL and functioning domains, a positive LS mean difference indicates higher QoL/functioning in the luspatercept group than placebo, whereas for all other domains, a positive LS mean difference indicates higher symptoms in the placebo group. BSC, best supportive care; EORTC QLQ-C30, European Organisation for Research and Treatment of Cancer’s Core Quality of Life Questionnaire; LS, least squares; MCID, minimal clinically important difference; SE, standard error.

**Table 4:** Baseline QOL-E scores.

QOL-E Domain ^a^	Baseline Score in MEDALIST Mean (SD) (N = 225)
Physical well-being	52.9 (21.5)
Functional well-being	53.7 (32.4)
Social and family life	48.4 (37.6)
Sexual well-being	62.4 (36.3)
Fatigue	75.0 (14.1)
MDS-specific disturbances	57.0 (23.7)
Treatment outcome index ^b^	54.7 (20.7)
General ^c^	58.7 (21.1)
All ^d^	58.1 (21.1)

^a^ Higher scores represent better QoL across all domains and summary scales. ^b^ Summary of physical well-being, functional well-being, and MDS-specific disturbances domain scores. ^c^ Summary of all domain scores except for MDS-specific disturbances. ^d^ Summary of all domain scores. MDS, myelodysplastic syndromes; QOL-E, Quality of Life Assessment in MDS questionnaire; QoL, quality of life; SD, standard deviation.

**Table 5:** LS mean difference in change in QOL-E domain scores from baseline to week 25 between luspatercept + BSC and placebo + BSC ^a^.

QOL-E Domain	LS Mean (SE) Difference ^b^ at Week 25	MCID
Physical well-being	−5.28 (3.18)	10.74
Functional well-being	−6.07 (4.63)	16.16
Social and family life	−8.70 (4.50)	18.76
Sexual well-being	0.31 (4.49)	18.08
Fatigue	−5.10 (2.03)	7.03
MDS-specific disturbances	−2.03 (3.01)	11.86
Treatment outcome index ^c^	−4.71 (2.80)	10.33
General ^d^	−6.30 (2.50)	10.51
All ^e^	−5.10 (2.70)	10.55

^a^ Data from longitudinal mixed-model analyses. ^b^ Differences in scores between luspatercept and placebo arms were considered clinically meaningful if the difference in the change from baseline between treatment arms exceeded the MCID threshold. MCID was defined as ≥0.5 standard deviations of the baseline score. ^c^ Summary of physical well-being, functional well-being, and MDS-specific disturbances domain scores. ^d^ Summary of all domain scores except for MDS-specific disturbances. ^e^ Summary of all domain scores. BSC, best supportive care; LS, least squares; MCID, minimal clinically important difference; MDS, myelodysplastic syndromes; QOL-E, Quality of Life Assessment in MDS questionnaire; SE, standard error.

**Table 6:** Item response on the QOL-E MDS-specific disturbances domain.

MDS-Specific Disturbances	Baseline, *n*/N (%)		Week 25, *n*/N (%)	
	Luspatercept + BSC	Placebo + BSC	Luspatercept + BSC	Placebo + BSC
Patients responding “Not at all”				
Being dependent on transfusions disturbs your daily life	22/147 (15.0)	19/74 (25.7)	48/108 (44.4)	11/52 (21.2)
Not being able to do housework disturbs your daily life	57/147 (38.8)	32/75 (42.7)	49/109 (45.0)	26/53 (49.1)
Not being able to travel either short or long distances disturbs your daily life	54/147 (36.7)	25/75 (33.3)	40/109 (36.7)	20/53 (37.7)
Being dependent on the hospital, doctors, and/or nurses disturbs your daily life	56/145 (38.6)	30/74 (40.5)	43/108 (39.8)	16/53 (30.2)
Stress and worry because of the illness disturb your daily life	41/149 (27.5)	19/75 (25.3)	33/108 (30.6)	18/53 (34.0)
Side-effects of the treatment disturb your daily life	91/143 (63.6)	48/74 (64.9)	57/106 (53.8)	32/53 (60.4)
Patients responding “Never”				
During the last week did shortness of breath disturb you?	36/147 (24.5)	12/76 (15.8)	19/107 (17.8)	11/53 (20.8)

## Data Availability

The data that support the findings of this study are available from the corresponding author upon reasonable request.

## Supplementary Materials

The following are available online at https://www.mdpi.com/article/10.3390/jcm11010027/s1, Figure S1: Observed mean change from baseline in patient-reported (A) Role functioning, (B) Cognitive functioning, (C) Social functioning, (D) Nausea/vomiting, (E) Pain, (F) Insomnia, (G) Appetite loss, (H) Constipation, (I) Diarrhea, and (J) Financial difficulties scores through Week 25 on the EORTC QLQ-C30. Figure S2: Observed mean change from baseline in patient-reported (A) Physical well-being, (B) Functional well-being, (C) Social and family life, (D) Sexual well-being, (E) Fatigue, (F) Treatment outcome index (summary of Physical well-being, Functional well-being, and MDS-specific disturbances domain scores), and (G) All (summary of all domain scores) scores through Week 25 on the QOL-E.
